# Real-world outcomes of first-line pembrolizumab plus pemetrexed-carboplatin for metastatic nonsquamous NSCLC at US oncology practices

**DOI:** 10.1038/s41598-021-88453-8

**Published:** 2021-04-28

**Authors:** Vamsidhar Velcheti, Xiaohan Hu, Bilal Piperdi, Thomas Burke

**Affiliations:** 1 NYU Langone, Perlmutter Cancer Center, 160 E 34th St, New York, NY 10016 USA; 2 Merck & Co., Inc., 2000 Galloping Hill Rd., Kenilworth, NJ 07033 USA; 3 Gilead Sciences, Morris Plains, NJ USA

**Keywords:** Non-small-cell lung cancer, Outcomes research, Cancer immunotherapy

## Abstract

Evidence from real-world clinical settings is lacking with regard to first-line immunotherapy plus chemotherapy for the treatment of non-small cell lung cancer (NSCLC). Our aim was to describe outcomes for patients treated with first-line pembrolizumab-combination therapy for metastatic nonsquamous NSCLC in US oncology practices. Using an anonymized, nationwide electronic health record-derived database, we identified patients who initiated pembrolizumab plus pemetrexed-carboplatin in the first-line setting (May 2017 to August 2018) after diagnosis of metastatic nonsquamous NSCLC that tested negative for *EGFR* and *ALK* genomic aberrations. Eligible patients had ECOG performance status of 0–1. An enhanced manual chart review was used to collect outcome information. Time-to-event analyses were performed using the Kaplan–Meier method. Of 283 eligible patients, 168 (59%) were male; median age was 66 years (range 33–84); and the proportions of patients with PD-L1 tumor proportion score (TPS) of ≥ 50%, 1–49%, < 1%, and unknown were 28%, 27%, 28%, and 17%, respectively. At data cutoff on August 31, 2019, median patient follow-up was 20.3 months (range 12–28 months), and median real-world times on treatment (rwToT) with pembrolizumab and pemetrexed were 5.6 (95% CI 4.5–6.4) and 2.8 months (95% CI 2.2–3.5), respectively. Median overall survival (OS) was 16.5 months (95% CI 13.2–20.6); estimated 12-month survival was 59.5% (95% CI 53.3–65.0); rwProgression-free survival was 6.4 months (95% CI 5.4–7.8); and rwTumor response rate (complete or partial response) was 56.5% (95% CI 50.5–62.4). Median OS was 20.6, 16.3, 13.2, and 13.7 months for patient cohorts with PD-L1 TPS ≥ 50%, 1–49%, < 1%, and unknown, respectively. These findings demonstrate the effectiveness of pembrolizumab plus pemetrexed-carboplatin by describing clinical outcomes among patients with metastatic nonsquamous NSCLC who were treated at US oncology practices.

## Introduction

Lung cancer is often diagnosed at advanced stages when surgical removal is not feasible; therefore, the choice of first-line systemic therapy is important^1,2^. The availability of immune checkpoint inhibitors of the programmed death 1 (PD-1) pathway, including pembrolizumab, nivolumab, atezolizumab, and durvalumab, has recently transformed the choices of systemic anticancer therapy for late-stage non-small cell lung cancer (NSCLC).

In the United States (US), the results of the phase 2 KEYNOTE-021G study^3^ led to accelerated approval on May 10, 2017, by the US Food and Drug Administration (FDA) of first-line pembrolizumab plus chemotherapy for advanced nonsquamous NSCLC. These results were further supported by subsequent findings of the randomized, placebo-controlled, double-blind KEYNOTE-189 trial, which indicated significant benefits of adding first-line pembrolizumab to standard chemotherapy of pemetrexed and a platinum-based drug for patients with metastatic nonsquamous NSCLC without epidermal growth factor receptor (*EGFR*) or anaplastic lymphoma kinase (*ALK*) mutations. Patients who received pembrolizumab plus pemetrexed-platinum experienced significantly longer overall survival (OS) and progression-free survival (PFS) as compared with those who received chemotherapy alone^4,5^. Survival benefits were recorded across PD ligand 1 (PD-L1) tumor expression categories^4–6^. Furthermore, a recently published pooled analysis of three clinical trials found clinically meaningful response and survival benefits and manageable safety for patients with PD-L1-negative advanced NSCLC, both nonsquamous and squamous, who received pembrolizumab plus chemotherapy versus chemotherapy alone^7^.

National Comprehensive Cancer Network (NCCN) guidelines currently list the combination of pembrolizumab, pemetrexed, and carboplatin or cisplatin as the category 1 preferred regimen for advanced/metastatic adenocarcinoma, large cell, and NSCLC not otherwise specified (NOS) in the first-line setting^1^. This regimen is recommended for patients with good performance status irrespective of tumor PD-L1 tumor proportion score (TPS), except in the presence of an oncogene predicting lack of benefit or other contraindication to PD-1/PD-L1 inhibitor therapy. Patient-reported outcomes data demonstrated improved global health status/quality of life at 21 weeks with pembrolizumab-chemotherapy compared with placebo-chemotherapy in KEYNOTE-189^8^.

Clinical trials, such as KEYNOTE-189, are designed to maximize internal validity by enrolling patients with adequate organ function, good performance status, and no select comorbidities, who then receive therapy in settings with predetermined care. Instead, practicing oncologists treat a broader, more heterogeneous patient population under clinical conditions that are often time- and resource-limited. Therefore, it is important to also understand the effectiveness and safety of medical interventions outside the clinical trial setting^9,10^. Well-conducted observational studies can provide this evidence to complement clinical trials^11^. In US oncology practices, the use of first-line immunotherapy for advanced NSCLC has increased rapidly since initial approvals in 2016^12^; however, data regarding real-world outcomes with first-line pembrolizumab-chemotherapy combination therapy in metastatic nonsquamous NSCLC are lacking.

Our aim was to evaluate clinical outcomes of first-line pembrolizumab plus pemetrexed-carboplatin in a real-world clinical setting for patients with good performance status (Eastern Cooperative Oncology Group performance status [ECOG PS] of 0 or 1) and tumor characteristics similar to those in KEYNOTE-189, namely, metastatic nonsquamous NSCLC without *EGFR* or *ALK* genomic aberrations. Our objectives were to determine real-world time on treatment (rwToT), OS, real-world PFS (rwPFS), and real-world tumor response rate (rwTRR) for patients receiving first-line pembrolizumab plus pemetrexed-carboplatin in routine clinical care.

## Results

### Patient population

**Figure 1:**
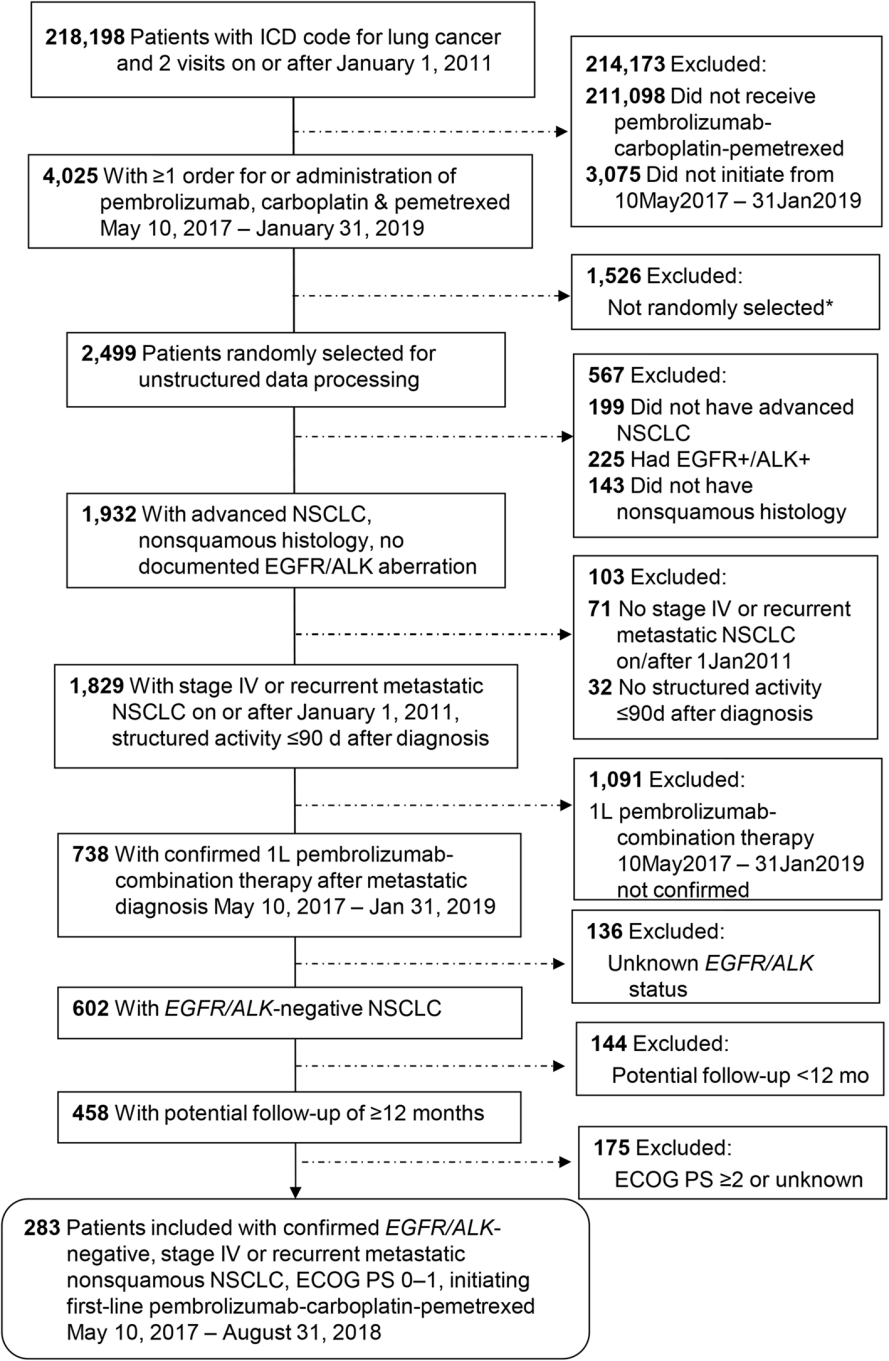
Patient selection from the Flatiron Health advanced NSCLC database. *1526 patients were excluded when the cohort of 2,499 patients was randomly selected from 4,025 patients for the application of further eligibility criteria via unstructured data processing (enhanced manual chart review). *ALK* anaplastic lymphoma kinase gene translocation, *ECOG* Eastern Cooperative Oncology Group performance status, *EGFR* epidermal growth factor receptor gene mutation, *ICD* international classification of diseases, *NSCLC* non-small cell lung cancer.

Of 738 patients with metastatic nonsquamous NSCLC who initiated first-line pembrolizumab-combination therapy on or after May 10, 2017, a total of 283 patients (38%) met the eligibility criteria for this analysis with tumors testing negative for *EGFR* and *ALK* genomic aberrations and ECOG PS of 0 or 1 (Fig. 1).

The median age overall was 66 years, and 21% of patients were 75 years or older; 59% of patients were male; and of 250 patients with known race, 185 were White (74%) and 32 were Black (13%; Table 1). Over 90% of patients received an initial NSCLC diagnosis at stage IV, and > 90% had a positive smoking history. Almost half of patients (46%) were located in the southern US; and all but 6 patients (2%) were treated at community oncology clinics. Twelve percent had a recorded history of brain metastasis (Table 1).

**Table 1:** Baseline patient characteristics.

	All patients, N = 283	PD-L1 expression level			
		≥ 50%, n = 79	1–49%, n = 77	< 1%, n = 79	Unknown, n = 48
Sex, male	168 (59.4)	48 (60.8)	42 (54.5)	48 (60.8)	30 (62.5)
Age, median (range)	66 (33–84)	68 (33–84)	66 (47–84)	65 (42–83)	66 (52–83)
**Age group**					
< 65 years	118 (41.7)	30 (38.0)	31 (40.3)	36 (45.6)	21 (43.8)
65–74 years	106 (37.5)	29 (36.7)	30 (39.0)	28 (35.4)	19 (39.6)
≥ 75 years	59 (20.8)	20 (25.3)	16 (20.8)	15 (19.0)	8 (16.7)
**Race, available data**^**a**^	250 (88.3)	69 (87.3)	68 (88.3)	70 (88.6)	43 (89.6)
White	185 (74.0)	50 (72.5)	53 (77.9)	49 (70.0)	33 (76.7)
Black or African American	32 (12.8)	7 (10.1)	9 (13.2)	11 (15.7)	5 (11.6)
Asian	5 (2.0)	1 (1.4)	1 (1.5)	2 (2.9)	1 (2.3)
Hispanic or Latino	1 (0.4)	1 (1.4)	0	0	0
Other	27 (10.8)	10 (14.5)	5 (7.4)	8 (11.4)	4 (9.3)
**Smoking status**					
History of smoking	260 (91.9)	72 (91.1)	72 (93.5)	73 (92.4)	43 (89.6)
No history of smoking	23 (8.1)	7 (8.9)	5 (6.5)	6 (7.6)	5 (10.4)
**Geographic region, available data**^**a**^	276 (97.5)	77 (97.5)	75 (97.4)	77 (97.5)	47 (97.9)
Midwest	41 (14.9)	10 (13.0)	10 (13.3)	14 (18.2)	7 (14.9)
Northeast	65 (23.6)	18 (23.4)	18 (24.0)	23 (29.9)	6 (12.8)
South	131 (47.5)	37 (48.1)	39 (52.0)	33 (42.9)	22 (46.8)
West	39 (14.1)	12 (15.6)	8 (10.7)	7 (9.1)	12 (25.5)
**Stage at diagnosis, available data**^**a**^	280 (98.9)	79 (100)	74 (96.1)	79 (100)	48 (100)
Stage IV	256 (91.4)	74 (93.7)	69 (93.2)	72 (91.1)	41 (85.4)
Brain metastasis, yes	34 (12.0)	11 (13.9)	10 (13.0)	9 (11.4)	4 (8.3)
**ECOG performance status**					
0	134 (47.3)	38 (48.1)	37 (48.1)	34 (43.0)	25 (52.1)
1	149 (52.7)	41 (51.9)	40 (51.9)	45 (57.0)	23 (47.9)
**Charlson comorbidity index**					
Mean (SD)	3.5 (3.2)	3.4 (3.3)	3.7 (3.2)	3.6 (3.3)	3.2 (3.1)
Median (range)	2 (0–11)	2 (0–9)	6 (0–10)	2 (0–11)	1.5 (0–10)

Data are n (%) unless otherwise noted. Percentages may not add up to 100 because of rounding.

*ECOG* Eastern Cooperative Oncology Group.

^a^Percentages for race, geographic region, and stage at diagnosis represent the percentages of patients with available data.

The proportions of patients were evenly distributed across PD-L1 TPS categories, with 28%, 27%, and 28% having a PD-L1 TPS ≥ 50%, 1–49%, and < 1%, respectively; 17% had unknown PD-L1 TPS. Patient characteristics were mostly similar across the PD-L1 expression cohorts (Table 1).

### Real-world time on treatment

At data cutoff (August 31, 2019), the median observed follow-up time from pembrolizumab initiation to data cutoff was 21.5 months (range 12–28), while median patient follow-up time from pembrolizumab initiation to the date of death or data cutoff was 20.3 months (range 12–28; Table 2).

**Table 2:** Real-world outcomes of first-line pembrolizumab plus pemetrexed-carboplatin.

Outcome	All patients, N = 283	PD-L1 expression level			
		≥ 50%, n = 79	1–49%, n = 77	< 1%, n = 79	Unknown, n = 48
Observed follow-up, median (range), mo	21.5 (12.1–27.5)	21.1 (12.3–27.3)	21.3 (12.3–27.5)	22.3 (12.1–27.5)	19.9 (12.5–26.4)
Patient follow-up, median (range), mo	20.3 (12.3–27.5)	19.4 (13.3–26.1)	20.4 (12.3–27.5)	22.5 (12.3–27.5)	19.5 (12.5–26.4)
**rwTumor response, n**^**a**^	160	51	42	41	26
rwTumor response rate, % (95% CI)	56.5% (50.5–62.4)	64.6% (53.0–75.0)	54.5% (42.8–65.9)	51.9% (40.4–63.3)	54.2% (39.2–68.6)
**rwProgression-free survival, n**	283	79	77	79	48
No. events (%)	221 (78.1)	58 (73.4)	64 (83.1)	65 (82.3)	34 (70.8)
Median in months (95% CI)	6.4 (5.4–7.8)	8.8 (7.2–11.6)	5.9 (4.6–8.1)	5.0 (4.3–6.6)	5.1 (3.2–12.3)
Rate at month 6 in % (95% CI)	52.7 (46.6–58.4)	66.5 (54.9–75.9)	49.4 (37.8–59.9)	45.6 (34.0–56.4)	46.6 (31.9–60.1)
Rate at month 12 in % (95% CI)	30.3 (24.9–35.8)	37.8 (27.0–48.4)	24.4 (15.4–34.4)	24.2 (15.1–34.6)	38.9 (24.7–52.9)
Rate at month 18 in % (95% CI)	20.3 (15.5–25.6)	26.2 (16.5–36.9)	16.2 (8.6–25.8)	15.1 (7.9–24.5)	27.8 (15.1–42.0)
**Overall survival, n**	282^b^	79	76	79	48
No. events (%)	152 (53.9)	39 (49.4)	44 (57.9)	44 (55.7)	25 (52.1)
Median in months (95% CI)	16.5 (13.2–20.6)	20.6 (14.8–NR)	16.3 (11.6–22.4)	13.2 (10.1–21.5)	13.7 (7.9–NR)
Rate at month 6 in % (95% CI)	75.4 (69.8–80.0)	80.7 (70.1–87.9)	76.0 (64.6–84.1)	73.8 (62.4–82.2)	68.0 (52.7–79.4)
Rate at month 12 in % (95% CI)	59.5 (53.3–65.0)	65.1 (53.3–74.5)	59.6 (47.5–69.8)	54.3 (42.2–64.8)	58.4 (42.7–71.2)
Rate at month 18 in % (95% CI)	48.6 (42.2–54.6)	54.6 (42.2–65.5)	47.5 (35.6–58.6)	46.0 (34.0–57.2)	43.6 (27.5–58.6)

Observed follow-up was defined as the time from pembrolizumab initiation to database cutoff. Patient follow-up was defined as time from pembrolizumab initiation to the date of death or data cutoff, whichever occurred first.

*Mo* month, *NR* not reached.

^a^Real-world tumor response (rwTR) was defined as complete response or partial response.

^b^One patient was excluded for negative OS value when setting the date of death to the 15th of the month.

**Figure 2:**
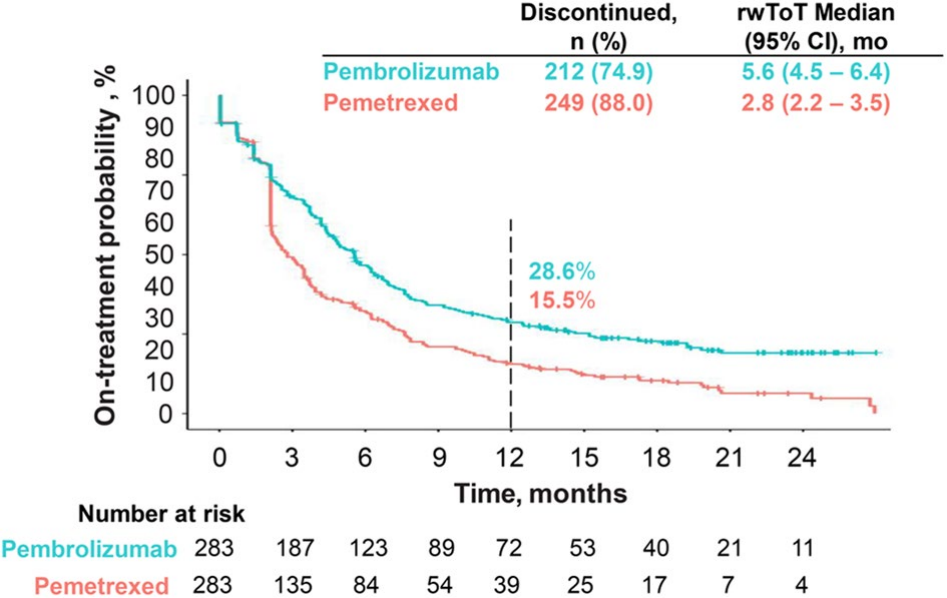
Kaplan–Meier curves depicting real-world time on treatment (rwToT) with pembrolizumab and pemetrexed for 283 patients with metastatic nonsquamous NSCLC who initiated first-line pembrolizumab plus pemetrexed-carboplatin.

The median rwToT with pembrolizumab was 5.6 months (95% CI 4.5–6.4), and the estimated 6- and 12-month on-treatment rates for pembrolizumab were 47% (95% CI 41–52%) and 29% (95% CI 23–34%), respectively (Fig. 2). For pemetrexed, the median rwToT was 2.8 months (95% CI 2.2–3.5) with 6- and 12-month on-treatment rates of 32% (95% CI 26–37%) and 16% (95% CI 12–20%), respectively. The restricted mean rwToT at 12 months for pembrolizumab and pemetrexed was 6.3 (95% CI 5.7–6.8) and 4.7 months (95% CI 4.3–5.2), respectively. The distribution of the number of cycles of pembrolizumab, pemetrexed, and carboplatin is summarized in Supplementary Table S1.

### Overall survival and real-world progression-free survival

**Figure 3:**
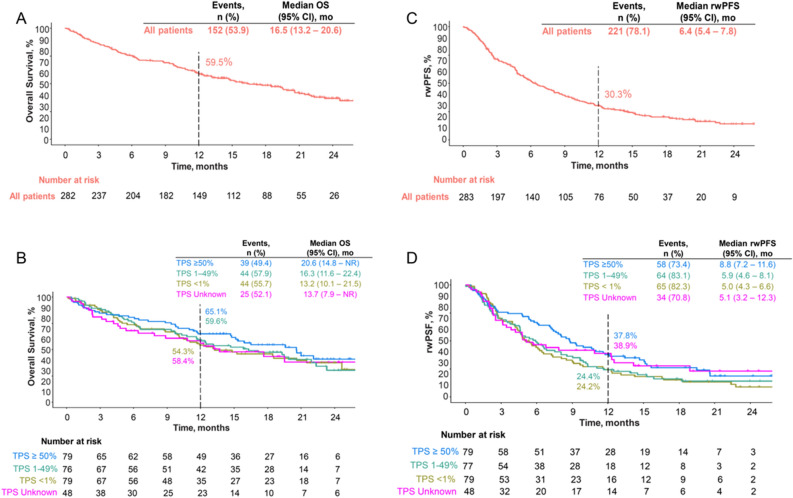
Overall survival (OS) and real-world progression-free survival (rwPFS): (**A**) OS for all patients, (**B**) OS by PD-L1 tumor proportion score, (**C**) rwPFS overall, (**D**) rwPFS by PD-L1 tumor proportion score.

The median OS for all patients was 16.5 months (95% CI 13.2–20.6), with an estimated survival rate of 59% at 12 months (Table 2). Patients with PD-L1 TPS ≥ 50% had a median OS of 20.6 months and a 12-month survival rate of 65%. Patients with PD-L1 TPS 1–49% or < 1% experienced median OS of 16.3 and 13.2 months, respectively, and 12-month survival rates of 60% and 54%, respectively (see Table 2). Figure 3 depicts the Kaplan–Meier curves overall and by PD-L1 category.

Twelve patients (4%) initiated first-line pembrolizumab-combination therapy > 90 days after the metastatic diagnosis date. There was no impact on OS overall or in PD-L1 TPS cohorts after excluding this group of patients (median OS, 16.5 months; 95% CI 13.2–20.6; see online Supplementary Table S2).

The median rwPFS overall was 6.4 months (95% CI 5.4–7.8). The median rwPFS in the PD-L1 ≥ 50% cohort was 8.8 months and ranged from 5.0 to 5.9 months in the other three PD-L1 TPS cohorts (Table 2; Fig. 3).

### Real-world tumor response

The overall rwTRR was 57% and was 65%, 55%, and 52% for patients with tumor PD-L1 TPS ≥ 50%, 1–49%, and < 1%, respectively (see Table 2).

Complete response was recorded as the best response for 21 patients (7%), including 14%, 5%, and 8% in cohorts with PD-L1 TPS ≥ 50%, 1–49%, and < 1%, respectively (Table 3). Approximately half of the patients experienced a partial response (139 patients; 49%), while 39 (14%) had stable disease, and 30 (11%) had progressive disease. There was no evaluable assessment for one-fifth of the patients (54; 19%), most of whom had no documented response in their EHRs (Table 3).

**Table 3:** Best real-world tumor response (rwTR) to first-line pembrolizumab plus pemetrexed-carboplatin.

rwTR category^a^	All patients, N = 283	PD-L1 expression level			
		≥ 50%, n = 79	1–49%, n = 77	< 1%, n = 79	Unknown, n = 48
Complete response (CR)	21 (7.4)	11 (13.9)	4 (5.2)	6 (7.6)	0
Partial response (PR)	139 (49.1)	40 (50.6)	38 (49.4)	35 (44.3)	26 (54.2)
Stable disease	39 (13.8)	8 (10.1)	10 (13.0)	14 (17.7)	7 (14.6)
Progressive disease (PD)	30 (10.6)	6 (7.6)	11 (14.3)	8 (10.1)	5 (10.4)
**No evaluable assessment**^**b**^	54 (19.1)	14 (17.7)	14 (18.2)	16 (20.3)	10 (20.8)
Indeterminate response	19 (6.7)	6 (7.6)	3 (3.9)	6 (7.6)	4 (8.3)
Pseudoprogression	12 (4.2)	2 (2.5)	8 (10.4)	1 (1.3)	1 (2.1)
Not documented	53 (18.7)	16 (20.3)	11 (14.3)	17 (21.5)	9 (18.8)

Data are n (%). Percentages may not add up to 100 because of rounding.

^a^For patients with multiple real-world tumor response (rwTR) assessments, the best response was used (CR > PR > stable disease > PD).

^b^Patients without an evaluable rwTR assessment are counted in the ‘no evaluable assessment’ response category and may be counted more than once.

### Reasons for pembrolizumab discontinuation and subsequent lines of therapy

Overall, 186 patients (66%) discontinued first-line pembrolizumab, most commonly because of disease progression (97 patients, 52%), as summarized in online Supplementary Table S3. The percentages of patients who discontinued in PD-L1 TPS ≥ 50%, 1–49%, < 1%, and unknown PD-L1 cohorts were 62%, 69%, 72%, and 56%, respectively, of whom 41%, 55%, 60%, and 52%, respectively, discontinued because of disease progression. Other common reasons for pembrolizumab discontinuation were adverse events related to therapy (18% of those discontinuing) and disease-related symptoms not due to pembrolizumab (9%).

A total of 71 patients remained on first-line treatment at data cutoff. Of the other 212 patients, 91 patients (43%) received at least one subsequent line of therapy after first-line pembrolizumab-combination therapy. The most common second-line therapies were combination therapies including a vascular endothelial growth factor (VEGF) inhibitor and single agent chemotherapy (Supplementary Table S4).

Among those 91 patients initiating a subsequent line of therapy, 32 (35%) initiated a third line (most commonly single agent chemotherapy or anti-VEGF-based combination therapy), 10 (11%) initiated a fourth line, and 2 patients (2%) initiated fifth and sixth lines of therapy.

## Discussion

In this retrospective study, we identified 283 patients with metastatic nonsquamous NSCLC without *EGFR* or *ALK* genomic aberrations, and with good performance status (ECOG PS of 0 or 1), who received first-line pembrolizumab plus pemetrexed-carboplatin at US oncology practices following regulatory approval in May 2017. At the time of data cutoff (August 31, 2019), median patient follow-up was 20.3 months. Patients experienced a median OS of 16.5 months, with an estimated 12-month overall survival rate of 59%. Findings from this study demonstrate the effectiveness of first-line pembrolizumab plus carboplatin-pemetrexed in treating metastatic nonsquamous NSCLC in the real-world setting.

The median OS reported in our study (16.5 months) was somewhat shorter than that recorded for clinical trial participants in KEYNOTE-189 who received first-line pembrolizumab plus pemetrexed and carboplatin/cisplatin (median 22.0 months)^5^. Although our study population was generally similar to the clinical trial population in terms of demographics, three key differences should be noted. First, the present study included a slightly older patient sample (58% vs. 52% ≥ 65 years old, respectively^4,5^). Second, to reflect the use of pembrolizumab-combination therapy in the real world, we did not specifically exclude patients with comorbid conditions that would potentially have led to exclusion from KEYNOTE-189 – the median Charlson comorbidity index score was 2 and ranged up to 11^13^. Third, because routine oncology care is not as tightly managed as care in clinical trials, the treatment frequency and length may not be strictly followed per labeled indications. Indeed, we found that patients tended to stop pemetrexed earlier than pembrolizumab despite no documented disease progression: two-thirds of patients continued pembrolizumab after 4 cycles versus fewer than half who continued pemetrexed after 4 cycles (Supplemental Table S1). In KEYNOTE-189, the mean treatment durations were similar for pembrolizumab and pemetrexed (mean [SD], 9.8 [7.8] months)^5^, whereas, in the present study, the median rwToT of pembrolizumab therapy was double that of pemetrexed (5.6 vs. 2.8 months), and the restricted mean rwToT at 12 months was also longer (6.3 vs. 4.7 months, respectively). Moreover, less than half of patients (43%) discontinuing first-line pembrolizumab combination therapy in this study initiated a subsequent treatment, which may have had an impact on patient survival in addition to the early stopping of pemetrexed. Some prior observational studies have similarly reported slightly inferior survival with immunotherapy for NSCLC as compared with clinical trial findings^14–16^. Many of these studies included a mix of patients with variable performance status and prior therapies. On the other hand, our findings for OS, rwPFS, and rwTRR being best at higher levels of PD-L1 expression were consistent with the pattern observed in KEYNOTE-189 for OS, PFS, and ORR^4,5^.

One strength of this study is that we utilized the well-regarded, geographically representative Flatiron Health database, which draws on EHRs from patients with cancer throughout the US and is designed to support oncologic research initiatives^11,12,17,18^. The use of enhanced manual chart review enabled the gathering of patient outcomes on disease progression and tumor response from unstructured data, such as physician notes and radiology reports, in addition to the single endpoint of death. This allowed a better understanding of treatment effect, as tumor response of advanced cancer is mainly induced by the primary anticancer treatment. It is worth noting that the “real-world response” (rwTR) event captured in this analysis is different from the standard approach for measuring tumor response in clinical trials, namely, Response Evaluation Criteria in Solid Tumors (RECIST), because of the retrospective nature of this analysis and the complexity of applying RECIST criteria to a real-world study across practices. However, the rwTR variable captures critical information on clinicians’ assessment of change in disease burden based on radiographic imaging. In a similar fashion, the “real-world progression” (rwP) endpoint signals the growth of tumor or worsening of disease as judged by the treating clinician, providing information on disease progression. In this study, we observed that the median rwPFS for all patients was 6.4 months, and over half of patients experienced either a complete or partial tumor response, supporting the conclusion of effectiveness based on OS results for pembrolizumab plus pemetrexed-carboplatin in this patient population.

Another strength is that we studied almost 300 patients across community oncology practices in the US since the regulatory approval of this regimen over a median follow-up time approaching 2 years. We required the 12-month minimum timeframe from treatment initiation to data cutoff to reduce the likelihood of bias from too short a follow-up time for events occurring.

The interpretation of the results from this analysis is also subject to some limitations. A key study limitation is that we did not include a control group prescribed pemetrexed-carboplatin; however, our findings suggest substantially better survival outcomes than in the era before the introduction of PD-1/PD-L1 inhibitors. In a prior study of a similar patient population, also utilizing the Flatiron Health database, Abernethy and colleagues^19^ reported a median OS of 10.0 months (95% CI 9.4–10.8) for 1578 patients with nonsquamous NSCLC without documented *EGFR* and *ALK* genomic aberrations who received first-line systemic therapy, most commonly carboplatin plus pemetrexed with or without bevacizumab. One distinction to note, however, is that, because of unavailable data at the time, their patient population was not restricted to those with good performance status and negative tests for *EGFR* and *ALK* genomic aberrations^19^.

Another limitation lies in the criteria applied in sample selection due to data availability constraints. The patient sample was identified using a small number of key eligibility criteria in line with the approved indication for this pembrolizumab-combination regimen, for example biomarker status and performance status. However, several exclusion criteria for KEYNOTE-189, such as symptomatic central nervous system metastases, were not assessable (we did not have information about pretreatment of brain metastases). Dates of death, set to the 15th of the month, were not precise, because only month and year were provided in line with data deidentification requirements. Other general limitations of retrospective observational studies that would apply to this study include possible errors in EHR data recording, as well as errors in data gathering from the manual chart review. In addition, the majority of patients were treated at community oncology centers; our findings may not reflect the outcomes of patients treated at academic centers^20^. Finally, as this was a descriptive study of a nonrandomized patient population with NSCLC and no inferential statistical analysis was conducted, no conclusions regarding causality can be drawn directly from this work. Nevertheless, survival of patients with late-stage cancer is mainly determined by the primary anticancer treatment they receive.

Further research is needed to study immunotherapy-combination regimens for larger cohorts of patients with both nonsquamous and squamous metastatic NSCLC in real-world settings and over longer periods of time. We plan longer follow-up of this study cohort and thus of a larger patient population with the addition of those excluded from the current analyses because of < 12 months’ follow-up. A study focused on patients with suboptimal performance status (ECOG PS ≥ 2), a potential predictor of poor prognosis^21^ and commonly encountered in clinical practice^12,22^, would also be of interest. In addition, further studies involving assessment of patients’ quality of life would also be of value to depict a comprehensive picture of real-world outcomes for patients with late-stage NSCLC receiving immunotherapy-combination regimens.

In conclusion, our findings provide evidence of the effectiveness associated with first-line pembrolizumab plus pemetrexed-carboplatin in US oncology clinics for patients with good performance status and metastatic nonsquamous NSCLC without *EGFR* or *ALK* genomic aberrations. Results from this study complement clinical trial data by describing clinical outcomes of first-line pembrolizumab-combination therapy in a more heterogeneous patient population treated in real-world oncology practice.

## Methods

### Data source and patients

The Flatiron Health database is an oncology-specific database incorporating electronic health record (EHR) data for more than 2.2 million patients with cancer seen at approximately 800 sites of care throughout the US^23^. The deidentified data, used frequently for pharmacoepidemiological research, includes both structured data as well as unstructured data, such as physicians’ notes and radiology reports, collected via technology-enabled abstraction, as previously described^11^.

We drew on the Flatiron Health advanced NSCLC database, which includes patients with at least two visits recorded in the database on or after January 1, 2011, who had advanced NSCLC that was diagnosed on or after January 1, 2011, or early-stage NSCLC with subsequent development into recurrent or progressive disease on or after January 1, 2011. From this database, patients were identified who initiated pembrolizumab plus pemetrexed-carboplatin (pembrolizumab-combination therapy) from the FDA accelerated approval date (May 10, 2017), through January 2019. A cohort of 2499 patients was then randomly selected from these patients for the application of further eligibility criteria via unstructured data processing (enhanced manual chart review).

Patients eligible for this retrospective study received pembrolizumab plus pemetrexed-carboplatin in the first-line setting after a pathologically confirmed diagnosis of metastatic (stage IV) nonsquamous NSCLC with negative tests for *EGFR* and *ALK* genomic aberrations. In addition, eligible patients had ECOG PS of 0 or 1 and a potential study follow-up of 12 months minimum, hence initiated first-line therapy on or before August 31, 2018. We excluded patients with unknown ECOG PS and those with no structured activity recorded in the database (e.g., visits, medication administrations and orders) within 90 days of the metastatic diagnosis.

Approval of the study protocol by the Copernicus Group Institutional Review Board was obtained before study initiation; this approval included a waiver of informed consent. The Flatiron Health data were deidentified, and provisions were in place to prevent reidentification. All research was performed in accordance with relevant guidelines and regulations.

### Outcome measures

The exposure of interest was first-line pembrolizumab plus pemetrexed-carboplatin initiated after a diagnosis of metastatic NSCLC. The outcomes of interest were rwToT, OS, rwPFS, rwTRR, and reasons for discontinuation of pembrolizumab.

The rwToT endpoint was determined using intravenous drug administration records and defined as the length of time from first to last administration dates before discontinuation of first-line therapy. As previously described^24^, discontinuation of therapy was defined as either initiation of the next line of therapy, patient death while on therapy, or a gap of ≥ 120 days between the last administration date and last contact date or data cutoff (August 31, 2019). Pembrolizumab and pemetrexed are recommended per their respective drug labels to be continued until disease progression, unacceptable toxicity, or (for pembrolizumab only) 35 cycles; therefore, rwToT was assessed for pembrolizumab and pemetrexed separately as the (date of the last dose—date of the first dose) + 1 day.

Survival was determined from the start date of pembrolizumab-combination therapy to the date of death, with data censoring for patients who were alive at the last contact date or data cutoff (August 31, 2019), whichever occurred first. The date of death was determined using the Flatiron Health mortality 2.0 endpoint, which has been validated against the National Death Index with a sensitivity of 91% and specificity of 96% in advanced NSCLC^25^. We set the date of death to the 15th of the month, as only month and year were provided in the Flatiron Health dataset.

Enhanced manual chart review was used to obtain rwP, rwTR, and reasons for pembrolizumab discontinuation from unstructured data in physician notes, radiology reports, pathology reports, biomarker reports, and other sources. Endpoints were denoted using the preceding ‘rw’ to distinguish them from the analogous prospectively determined endpoints.

We defined rwPFS as the time from pembrolizumab-combination initiation to the first documented disease progression (rwP) event or death from any cause, whichever occurred first, with censoring of data from patients without rwP at the last recorded clinic encounter or initiation of a subsequent line of therapy, whichever occurred first. Chart abstractors identified the first episode of rwP as a distinct episode in which the treating clinician concluded that there had been growth or worsening of NSCLC, occurring at least 14 days after initiation of pembrolizumab-combination therapy, as previously detailed^17,18^.

In addition, chart abstractors captured the treating clinician’s assessment or interpretation of imaging tests and mapped each recorded tumor response (rwTR) to the categories of complete response (CR), partial response (PR), stable disease, progressive disease (PD), or other (indeterminate, pseudoprogression, not documented)^17^. The number and proportion of patients who had a CR or a PR was used to determine rwTRR. For patients with multiple rwTR assessments, the best response was used (CR > PR > stable disease > PD).

The reasons for discontinuation of pembrolizumab were also determined during the chart review and were recorded as adverse event related to therapy, progression, disease-related symptoms not due to therapy, completed treatment, financial reasons, patient request, no evidence of disease, unknown, and other.

### Statistical analyses

Descriptive analyses were conducted for patient characteristics, rwTR and reasons for pembrolizumab discontinuation. We calculated the observed follow-up duration as time from pembrolizumab initiation to database cutoff, in addition to the patient follow-up duration, calculated as time from pembrolizumab initiation to the date of death or data cutoff, whichever occurred first.

The Kaplan–Meier method was used for analysis of rwToT, OS, and rwPFS (the latter excluded pseudoprogression). The restricted mean rwToT at 12 months was also determined, as previously described^24^. In addition, a sensitivity analysis of OS was carried out by excluding patients who initiated first-line pembrolizumab-combination therapy > 90 days after the metastatic diagnosis date.

Analyses were conducted across all patients, as well as stratified by the PD-L1 TPS category (≥ 50%, 1–49%, < 1%, and unknown). A formal calculation of sample size and statistical power was not performed because of the exploratory nature of the study. Statistical analyses were conducted using the R language for statistical computing, version 3.6.3 (R Core Team, 2020; http://www.R-project.org/).

## Supplementary Information

Supplementary information.

## Data Availability

The data that support the findings of this study have been originated by Flatiron Health, Inc. These de‐identified data may be made available upon request and are subject to a license agreement with Flatiron Health; interested researchers should contact DataAccess@flatiron.com to determine licensing terms.
