# Supplementary material for: Real-world outcomes of first-line pembrolizumab plus pemetrexed-carboplatin for metastatic nonsquamous NSCLC at US oncology practices
Source: Sci Rep. 2021 Apr 28;11:9222. doi: 10.1038/s41598-021-88453-8 (PMC8080779; doi:10.1038/s41598-021-88453-8)
Supplement: Supplementary file 1 — Supplementary information. [file 41598_2021_88453_MOESM1_ESM.pdf]

Supplementary material

Real-world outcomes of first-line pembrolizumab plus pemetrexed-carboplatin for metastatic nonsquamous NSCLC at US oncology practices

Vamsidhar Velcheti[1,*], Xiaohan Hu[2], Bilal Piperdi[2], Thomas Burke[2]

[1]*NYU Langone, Perlmutter Cancer Center, 160 E 34th St, New York, NY 10016, USA*
[2]*Merck & Co., Inc., 2000 Galloping Hill Rd., Kenilworth, NJ 07033, USA*

Supplementary Table S1. Distribution of the number of pembrolizumab, pemetrexed, and carboplatin cycles administered

Number of cycles	Pembrolizumab	Pemetrexed	Carboplatin
1	27 (9.5)	26 (9.2)	27 (9.5)
2	19 (6.7)	18 (6.4)	18 (6.4)
3	23 (8.1)	25 (8.8)	26 (9.2)
4	23 (8.1)	78 (27.6)	148 (52.3)
5	10 (3.5)	14 (4.9)	22 (7.8)
≥6	181 (64.0)	122 (43.1)	42 (14.8)

Data are n (%). Percentages may not add up to 100 because of rounding.

Supplementary Table S2. Overall survival (OS) after excluding 12 patients who initiated first-line pembrolizumab plus pemetrexed-carboplatin >90 days after the metastatic diagnosis date

Survival	All patients	PD-L1 expression level			
		≥50%	1–49%	<1%	Unknown
	N = 271	n = 77	n = 73	n = 74	n = 47
No. events (%)	146 (53.9)	39 (50.6)	42 (57.5)	41 (55.4)	24 (51.1)
Median OS in months (95% CI)	16.5 (13.2–20.6)	20.6 (14.8–NA)	16.3 (10.8–22.4)	13.2 (10.1–21.5)	13.7 (7.9–NA)
Rate at month 6 in % (95% CI)	74.7 (69–79.5)	78.9 (67.9–86.5)	73.6 (61.8–82.3)	73.3 (61.4–82.1)	69.6 (54.1–80.8)
Rate at month 12 in % (95% CI)	58.9 (52.6–64.6)	64.1 (52.2–73.8)	57.8 (45.5–68.3)	53.8 (41.3–64.7)	57.2 (41.3–70.3)
Rate at month 18 in % (95% CI)	48.4 (41.9–54.6)	53.6 (41–64.6)	48.2 (36–59.5)	44.8 (32.4–56.4)	44.6 (28.2–59.7)

Supplementary Table S3. Reasons for discontinuation of first-line pembrolizumab therapy

		PD-L1 expression level			
	All patients N = 283	≥50% n = 79	1–49% n = 77	<1% n = 79	Unknown n = 48
Discontinued, n (%)	186 (65.7)	49 (62.0)	53 (68.8)	57 (72.2)	27 (56.2)
Reasons for discontinuation, n (%)*					
Progression	97 (52.2)	20 (40.8)	29 (54.7)	34 (59.6)	14 (51.9)
Adverse events related to therapy	34 (18.3)	12 (24.5)	10 (18.9)	8 (14.0)	4 (14.8)
Disease-related symptoms not due to therapy	17 (9.1)	4 (8.2)	3 (5.7)	5 (8.8)	5 (18.5)
Patient request	9 (4.8)	3 (6.1)	4 (7.5)	2 (3.5)	0
No evidence of disease	3 (1.6)	1 (2.0)	0	1 (1.8)	1 (3.7)
Completed treatment	2 (1.1)	0	1 (1.9)	1 (1.8)	0
Financial	2 (1.1)	0	1 (1.9)	1 (1.8)	0
Other	23 (12.4)	8 (16.3)	6 (11.3)	7 (12.3)	2 (7.4)
Unknown	2 (1.1)	1 (2.0)	0	0	1 (3.7)

*Patients could have more than one reason for discontinuation.

Supplementary Table S4. Distribution of subsequent lines of therapy

	All patients N = 212
**Second-line systemic therapy**	**91 (42.9)**
Anti-VEGF-based combination therapy	28 (13.2)
Single agent chemotherapy	25 (11.8)
PD-1/PD-L1 monotherapy	10 (4.7)
Platinum-based chemotherapy combination (without VEGF)	9 (4.2)
PD-1/PD-L1-based combination therapy	8 (3.8)
Non-platinum-based chemotherapy combination (without VEGF)	3 (1.4)
Clinical study drug based monotherapy	3 (1.4)
ALK inhibitor monotherapy	2 (0.9)
Anti-VEGF monotherapy	1 (0.5)
Clinical study drug based combination therapy	1 (0.5)
Other therapies	1 (0.5)
**Third-line systemic therapy**	**32 (15.1)**
Single agent chemotherapy	13 (6.1)
Anti-VEGF-based combination therapy	11 (5.2)
Non-platinum-based chemotherapy combination (without VEGF)	2 (0.9)
PD-1/PD-L1 monotherapy	2 (0.9)
ALK inhibitor monotherapy	2 (0.9)
PD-1/PD-L1-based combination therapy	1 (0.5)
Platinum-based chemotherapy combination (without VEGF)	1 (0.5)
**Fourth-line systemic therapy**	**10 (4.7)**
Single agent chemotherapy	7 (3.3)
Anti-VEGF-based combination therapy	1 (0.5)
PD-1/PD-L1-based combination therapy	1 (0.5)
PD-1/PD-L1 monotherapy	1 (0.5)
**Fifth-line systemic therapy**	**2 (0.9)**
Platinum-based chemotherapy combination (without VEGF)	1 (0.5)
Anti-VEGF-based combination therapy	1 (0.5)
**Sixth-line systemic therapy**	**2 (0.9)**
PD-1/PD-L1 monotherapy	1 (0.5)
Single agent chemotherapy	1 (0.5)

Data are n (% of all patients no longer on first-line therapy at data cutoff [n=212]).
ALK, anaplastic lymphoma kinase, PD-1/PD-L1, programmed death 1/programmed death ligand-1 inhibitor; VEGF, vascular endothelial growth factor.
